# Plaque-Adapted Virtual Monoenergetic Image Selection for Carotid Plaque Visualization Using Photon-Counting CT

**DOI:** 10.3390/diagnostics16172774

**Published:** 2026-08-28

**Authors:** Mirela Kostadinova, Lea B. Uebelacker, Tommaso D’Angelo, Giuseppe M. Bucolo, Ibrahim Yel, Vitali Koch, Leon D. Gruenewald, Scherwin Mahmoudi, Andreea-Ioana Nica, Leona S. Alizadeh, Aynur Goekduman, Hanns L. Kaatsch, Stephan Waldeck, Katrin Eichler, Thomas J. Vogl, Christian Booz, Daniel Overhoff

**Affiliations:** 1 Clinic for Radiology and Nuclear Medicine, University Hospital Frankfurt, Goethe University, 60590 Frankfurt am Main, Germany; 2 Diagnostic and Interventional Radiology Unit, BIOMORF Department, University Hospital “Policlinico G. Martino”, Via Consolare Valeria 1, 98100 Messina, Italy; 3 Department of Radiology and Neuroradiology, Bundeswehr Central Hospital, Rübenacher Straße 170, 56072 Koblenz, Germany

**Keywords:** carotid arteries, CT angiography, plaque composition, virtual monoenergetic imaging, photon-counting CT, dual-energy CT

## Abstract

**Objectives:** The objective of this study was to evaluate the impact of virtual monoenergetic image (VMI) reconstructions derived from photon-counting computed tomography (PCCT) on the assessment of carotid arteries, with a focus on optimizing keV selection based on plaque composition. **Methods:** This retrospective study included 111 patients (mean age 80 ± 7.5 years; 64 men; 47 women) with carotid sclerosis who underwent PCCT between April 2022 and February 2023. One lesion was analyzed per patient, each containing both calcified and non-calcified components. Quantitative measurements were performed in calcified plaque across energy levels from 40 to 120 keV and comprised attenuation, signal-to-noise ratio (SNR), contrast-to-noise ratio (CNR), corrected image noise (CIN) and artifact index (AIX). Two radiologists independently rated image quality, artifacts, and diagnostic assessability of both components using five-point scales. **Results:** Attenuation and CNR were highest at 40 to 50 keV, where CIN and AIX were also greatest. SNR followed a U-shaped course, lowest at 90 keV and highest at 110 to 120 keV, where intraluminal attenuation was too low for reliable luminal delineation. Reader ratings were highest at 40 to 50 keV for non-calcified components and at 70 to 80 keV for calcified plaque, with good interobserver agreement throughout (κ 0.74 to 0.92). After correction for multiple testing, adjacent energy levels were frequently indistinguishable. **Conclusions:** Optimal PCCT VMI energy levels depend on plaque composition. The findings support implementing standardized protocols with automatic dual-range reconstructions (40–50 keV and 70–80 keV) to enable efficient, individualized carotid plaque assessment in clinical practice.

## 1. Introduction

Stroke is the second leading cause of mortality worldwide and a major contributor to long-term disability [1,2]. Carotid artery stenosis is a critical risk factor, accounting for 10–20% of ischemic stroke cases [3,4,5]. Accurate imaging is imperative for the diagnosis and characterization of carotid stenoses, as it guides clinical decisions, particularly regarding surgical or endovascular intervention [6]. A precise evaluation of luminal narrowing and plaque characteristics is essential for optimal patient management [7,8].

CT angiography (CTA) is a pivotal modality for evaluating carotid artery disease due to its rapid acquisition, broad availability, and high spatial resolution [9]. However, conventional CTA is limited in the presence of heavy arterial calcifications, where blooming artifacts can obscure the lumen and lead to inaccurate stenosis measurements [10]. These inaccuracies can have substantial clinical consequences. Overestimation may lead to unwarranted interventions, such as carotid endarterectomy or stenting, which can result in adverse outcomes including cranial nerve injury, myocardial infarction, or perioperative stroke [11,12]. Conversely, underestimation may delay essential treatment, thereby increasing the risk of stroke recurrence in symptomatic individuals [13]. 

Recent advancements in CT technology have introduced photon-counting CT (PCCT) as a promising imaging modality [14,15,16]. Unlike conventional energy-integrating detectors, PCCT employs photon-counting detectors to improve spatial resolution, reduce image noise, and enhance material differentiation [17,18]. A notable advantage of PCCT is its ability to generate virtual monoenergetic images (VMI) at selectable energy levels, enhancing contrast and reducing artifacts, which is particularly valuable for vascular imaging [19].

Studies have demonstrated that low-energy VMI (40–55 keV) significantly enhances vascular contrast and image quality, facilitating better delineation of vascular structures, even with calcifications, and potentially allowing for reduced contrast media dose [20,21,22]. In photon-counting CT of the carotid arteries, non-calcified plaque has been shown to be most favorably depicted in this range [22]. While low-keV VMI improves visualization of non-calcified plaques and contrast enhancement, it also increases artifacts from calcifications. Higher keV level reconstructions have the potential to reduce these artifacts; however, their diagnostic value remains less well characterized, and the concept of tailoring keV selection to plaque composition, although previously proposed, has not been examined across the full energy range in the carotid arteries.

The present study is exploratory and extends these findings to the full virtual monoenergetic range up to 120 keV, with quantitative measurements confined to calcified plaque and reader assessment of both the calcified and the non-calcified component of each lesion. We hypothesized that the optimal energy level varies by plaque composition and that the commonly recommended low-keV range (40–55 keV) may not be optimal for heavily calcified arteries, underscoring the necessity for a more differentiated keV approach. 

## 2. Materials and Methods

The local institutional review board approved this retrospective study and waived the requirement to obtain written informed consent. 

### 2.1. Study Design and Population

**Figure 1:**
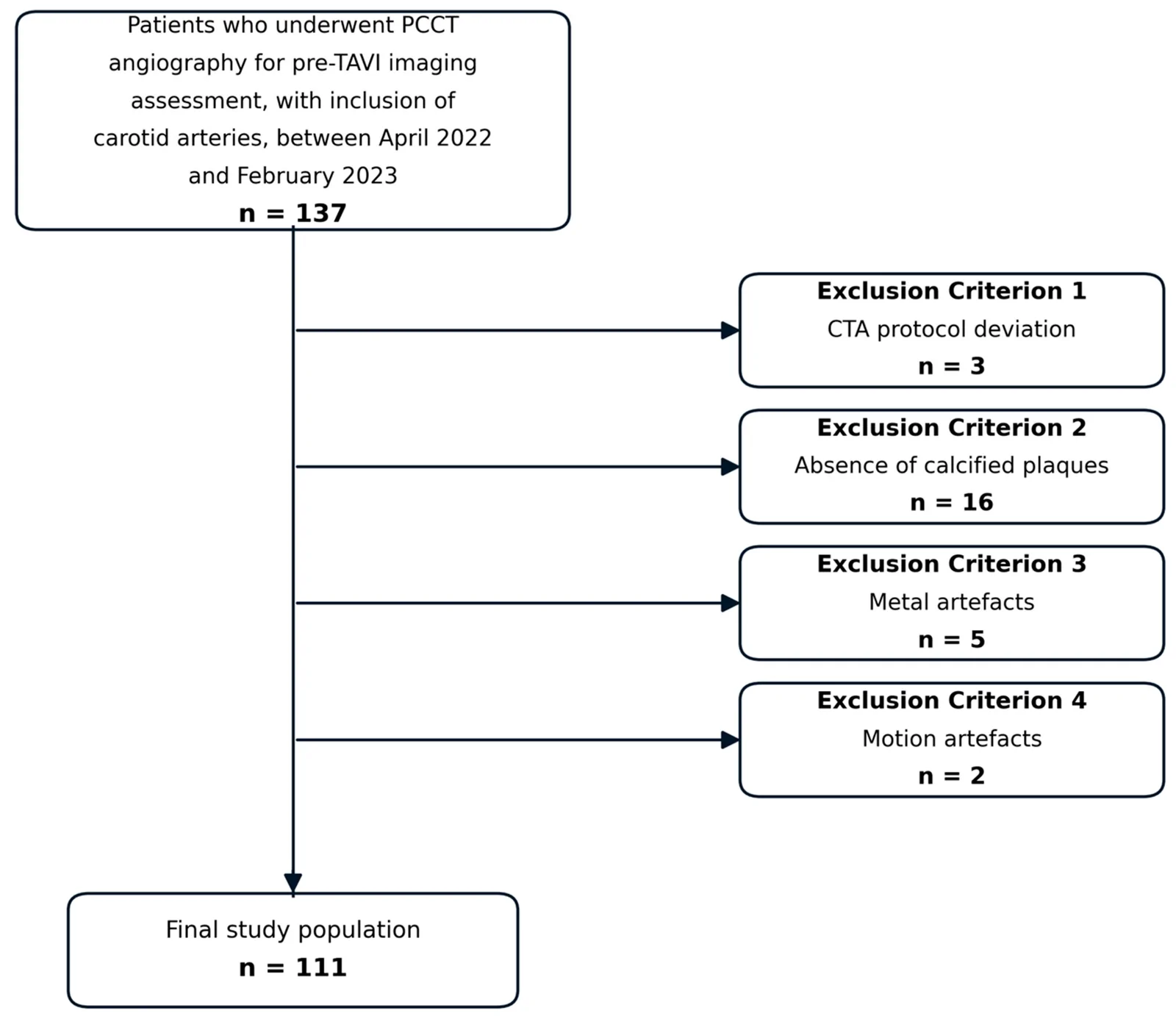
Flow chart showing the selection process in this study.

This retrospective study included 111 patients (64 men [57.7%], 47 women [42.3%]; mean age, 80 ± 7.5 years; mean BMI, 28 ± 5 kg/m^2^) with carotid sclerosis at the level of the carotid bifurcation who underwent PCCT between April 2022 and February 2023. The inclusion criteria encompassed pre-TAVI CTA protocols involving carotid imaging. Patients were excluded on the basis of protocol deviations (*n* = 3), poor image quality from metal or motion artifacts (*n* = 7), or the absence of calcified carotid plaques (*n* = 16). Both the presence of obscuring artifacts and the absence of calcified plaque were determined visually on the conventional polyenergetic and non-contrast images, before any virtual monoenergetic series were reviewed. Patients without a calcified plaque were excluded because ΔHU and the artifact index are defined relative to a hyperdense artifact radiating from a calcified lesion. One lesion in one carotid artery was analyzed per patient, and in all included patients this lesion contained both a calcified and a non-calcified component. Twenty-two of the 111 included patients (19.8%) were also part of the cohort reported in a previous study by our group [22]. The patient selection process is summarized in Figure 1, and characteristics are presented in Table 1.

### 2.2. CT Protocol

CT scans were performed on a dual-source PCCT scanner (NAEOTOM Alpha; Siemens Healthineers, Forchheim, Germany). Patients were positioned in a supine position with their arms at their sides. Preliminary scout images were acquired in both anteroposterior and lateral directions, covering the region from the neck to the iliac vessels. All examinations were performed in the craniocaudal scan direction. The study protocol commenced with an ECG-triggered non-contrast acquisition extending from the skull base to the proximal thigh, thereby including both carotid bifurcations. Contrast agent (Xenetix 350 mgI/mL; Guerbet, Villepinte, France) was then administered for the angiographic phase. The power injector (CT Motion; Ulrich Medical, Ulm, Germany) was utilized to administer 80 mL of contrast medium into a superficial forearm vein at 5 mL/s through a 20-gauge cannula, followed by a 50 mL saline flush at the same rate. The angiographic phase was initiated using bolus tracking, with the region of interest placed in the descending aorta, a threshold of 100 HU, and a delay of 5 s. An ECG-synchronized CTA was then acquired from the carotid arteries through the thorax and abdomen to the femoral arteries and triggered automatically at a predefined point. All acquisition and reconstruction parameters are summarized in Table 2. 

### 2.3. Image Reconstruction

All data were transmitted to a dedicated postprocessing workstation (syngo.via VB60A; Siemens Healthineers, Forchheim, Germany). Spectral postprocessing datasets were reconstructed with a slice thickness and increment of 1.0 mm using a soft tissue convolution kernel (Qr40). VMI series were reconstructed at virtual energy levels ranging from 40 to 120 keV in 10 keV increments using dedicated postprocessing software, and multiplanar reformations (axial and sagittal) were generated for each energy level.

### 2.4. Plaque Component Identification

Within each lesion, the calcified and non-calcified components were identified independently by the two reading radiologists (6 and 10 years of experience) on axial images of the conventional (120 kVp-equivalent) reconstructions of the non-contrast and contrast-enhanced acquisitions. Calcification was identified primarily by its visual appearance, with attenuation measured where the distinction was not immediately apparent. In accordance with established CT criteria, it was defined as material exceeding 130 HU [23,24], and all tissue below this threshold was regarded as non-calcified. No further subdivision of the non-calcified component was attempted, since the distinction relevant to energy-level selection is whether a lesion produces calcium-related blooming rather than the nature of its non-calcified content. A third reader (12 years of experience) was available to adjudicate disagreements, but the two readers identified the same components in all lesions and no adjudication was required. All analyzed lesions contained both a calcified and a non-calcified component, and each component was rated separately at every energy level, so no lesion-level categorization was performed.

### 2.5. Quantitative Image Analysis

Quantitative measurements were performed by one reader (6 years of experience). One lesion in one carotid artery was analyzed per patient. In patients with disease at both bifurcations, the lesion with the greatest plaque burden in which the residual lumen remained visible was selected, since a region of interest cannot be placed where the lumen is obliterated. A circular region of interest (ROI) was positioned intravascularly within the most hyperdense artifact radiating from the calcified plaque and in a non-affected area of the same artery, and intramuscularly in the adjacent neck musculature. Each ROI was drawn as large as the target structure permitted while remaining entirely within it and avoiding the borders, giving areas of 2 to 25 mm^2^. Where the residual lumen was narrow, the area was reduced accordingly, and the identical area was then used for the corresponding reference measurement in the same patient, so that signal and noise were sampled over comparable areas within each measurement pair. To ensure measurement consistency and reproducibility, ROI placement was repeated five times, and the average values of the measurements were used for statistical analyses. In the VMIs, the ROIs remained consistent across all energy levels due to the automated processing. Attenuation (in Hounsfield units, HU) and standard deviation (SD), taken as image noise, were recorded for each ROI. An additional ROI was placed in non-affected subcutaneous fat to calculate the corrected image noise (CIN = SD − SDfat), and attenuation and SD of non-affected muscle tissue were recorded to evaluate the signal-to-noise ratio (SNR = HUartery/SDfat) and contrast-to-noise ratio (CNR = (HUartery-HUmuscle)/SDfat). The corrected artifact burden (∆HU = HUartifact—HUno-artifact) and the artifact index (AIX = √(SD2artifact − SD2no-artifact)) were calculated as described in previous studies [25,26].

### 2.6. Qualitative Image Analysis

Two board-certified radiologists (with 6 and 10 years of experience in vascular imaging), independently and blinded to clinical data, evaluated VMI reconstructions from 40 to 120 keV. All series were anonymized, and the energy level was not shown in the series description. Series were presented to each reader in a separately generated randomized order. No washout interval was interposed between energy levels, as this would have required nine separate reading sessions for every patient. 

Readers rated the overall image quality, artifact burden, and diagnostic assessability separately for calcified and non-calcified plaque using three five-point Likert scales. The scales ranged from 1 = poor to 5 = excellent for image quality, from 1 = extensive artifacts to 5 = no artifacts for artifact presence, and from 1 = non-diagnostic to 5 = excellent for diagnostic assessability. The 10 keV increment was chosen so that perceptible differences between energy levels could be discerned while avoiding subtle changes introduced by closely spaced thresholds. Each reader could freely adjust window settings and scroll through the entire image stack, including multiplanar reformations. Ratings were averaged across the two readers to derive the final scores.

### 2.7. Statistical Analysis

Statistical analysis was performed using SPSS version 27 (IBM Corporation, Armonk, NY, USA). One lesion in one carotid artery was analyzed per patient, so that each patient contributed a single set of measurements at each energy level. The five repeated region-of-interest placements were averaged before analysis.

Data distribution was evaluated using the Shapiro–Wilk test. As the quantitative parameters were not normally distributed, the comparison of values between each VMI reconstruction was assessed using the Friedman test. Qualitative ratings, being ordinal, were likewise compared between energy levels using the Friedman test, separately for calcified and non-calcified plaque components. In both analyses, post hoc pairwise comparisons between energy levels were performed with the Wilcoxon signed-rank test and adjusted for multiple testing using the Holm–Bonferroni procedure.

Quadratically weighted Cohen’s kappa was used to assess the reliability of agreement between the two readers at each energy level. A kappa statistic between 0.61 and 0.8 was rated as substantial agreement, and above 0.81 as almost perfect agreement. A kappa of 0 meant poor agreement; between 0 and 0.2 meant slight agreement; 0.21–0.4 meant fair agreement; and 0.41–0.6 meant moderate agreement [27].

The statistically significant difference was indicated by a *p*-value less than 0.05, and all statements of significance refer to adjusted values. All continuous variables are shown as mean and standard deviation (SD). 

## 3. Results

### 3.1. Quantitative Image Analysis

Quantitative analyses are based on 111 lesions in 111 patients, one lesion per patient. The mean cumulative CT dose index (CTDIvol) across all examinations was 6.12 ± 2.18 mGy, and the mean dose-length product was 412.6 ± 194.5 mGy·cm.

**Figure 2:**
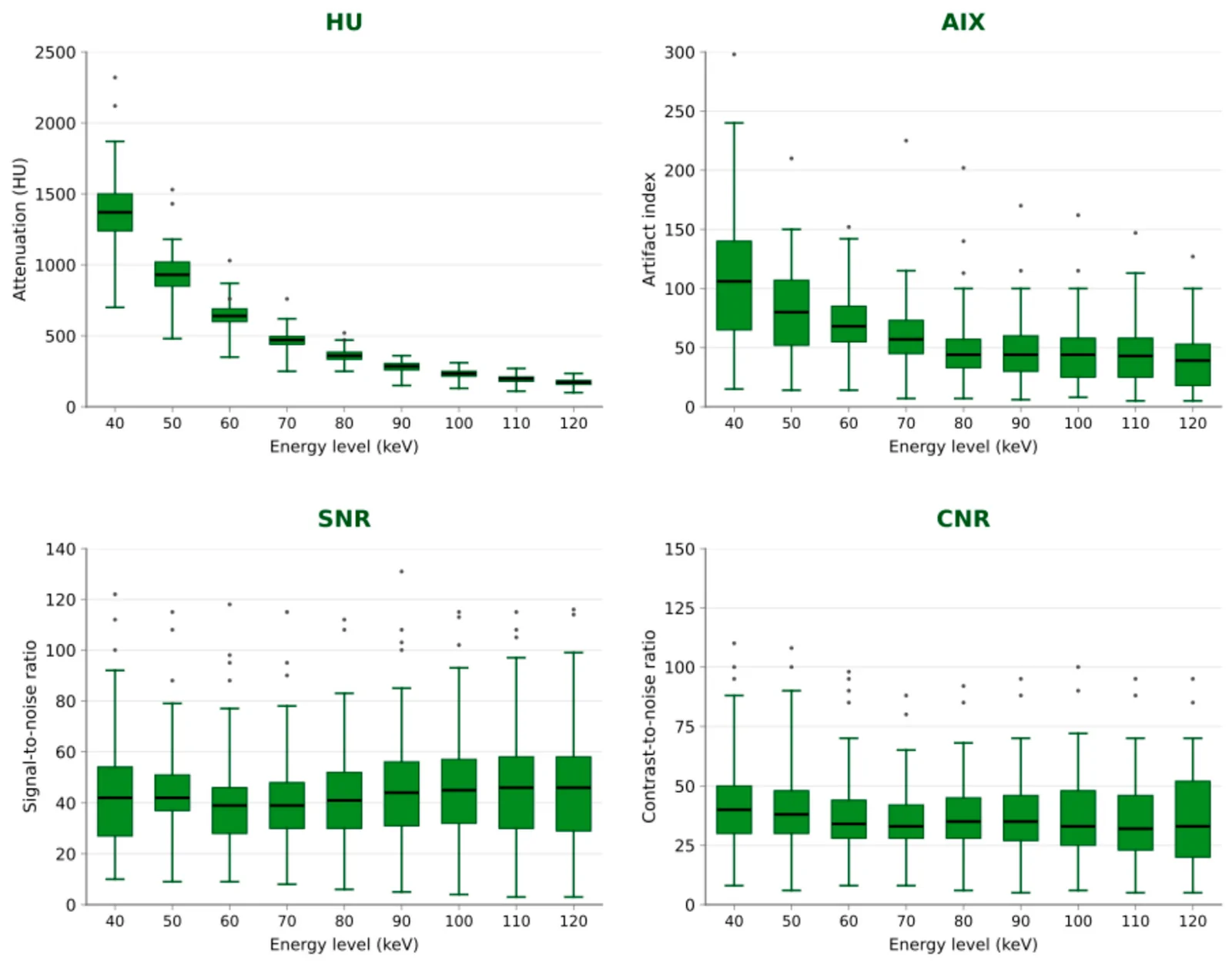
Distribution of attenuation (HU), artifact index (AIX), signal-to-noise ratio (SNR), and contrast-to-noise ratio (CNR) across virtual monoenergetic image (VMI) reconstructions from 40 to 120 keV. HU, AIX, and CNR peaked at 40 keV, whereas SNR followed a U-shaped course with a nadir at 90 keV and its highest values at 120 keV. Dots indicate individual observations beyond the whiskers. HU, Hounsfield units; AIX, artifact index; SNR, signal-to-noise ratio; CNR, contrast-to-noise ratio.

Attenuation values were highest in the 40 keV VMI series compared with all other VMI reconstructions, decreasing progressively as keV increased (*p* < 0.0001). At 40 keV, the mean intraluminal attenuation in the carotid arteries was 1387.10 ± 70.55 HU, declining to 172.3 ± 9.4 HU at 120 keV. The AIX followed the same trend, with significantly higher values at 40 keV (112.46 ± 99.07) than at 120 keV (38.96 ± 34.29; *p* < 0.0001). CNR and CIN were likewise highest at lower keV (42.00 ± 21.91 and 76.14 ± 91.98, respectively, at 40 keV) compared with 36.80 ± 52.41 and 31.81 ± 33.16 at 120 keV. SNR did not vary monotonically with energy. It decreased from 42.58 ± 21.55 at 40 keV to its lowest value of 37.84 ± 21.47 at 90 keV, with a local increase at 80 keV (41.16 ± 18.91), and then rose to its highest values at 110 and 120 keV (45.59 ± 23.47 and 46.05 ± 24.83) (Figure 2). A detailed overview of all quantitative parameters across the evaluated keV levels is provided in Table 3.

### 3.2. Qualitative Image Analysis

For calcified plaque, the highest ratings were obtained at 70 and 80 keV, with an overall image quality of 4.4 ± 0.5 at both levels, artifact ratings of 4.3 ± 0.6 and 4.5 ± 0.5, and a diagnostic assessability of 4.2 ± 0.7 at both. At 40 and 50 keV, the corresponding values were lower, with an overall image quality of 3.8 ± 0.7 at both levels and a diagnostic assessability of 3.5 ± 0.9 and 3.7 ± 0.8.

For non-calcified components, the highest ratings were obtained at 40 and 50 keV, with an overall image quality of 4.4 ± 0.5, artifact ratings of 4.2 ± 0.7, and a diagnostic assessability of 4.4 ± 0.5 at both levels.

Interobserver agreement was highest at the same energy levels that received the highest ratings, with kappa values of 0.91 (95% CI 0.87–0.95) at 70 keV and 0.92 (0.88–0.96) at 80 keV for calcified plaque, and 0.88 (0.82–0.93) at 40 keV and 0.89 (0.84–0.93) at 50 keV for non-calcified components. Across all energy levels, agreement ranged from 0.74 to 0.92, corresponding to substantial to almost perfect agreement. 

Qualitative ratings varied significantly over the energy range as a whole for all scales. All Friedman tests gave *p* < 0.001, with the single exception of artifact ratings in non-calcified components (*p* = 0.021), where ratings varied by only 0.2 points across the entire energy range. The post hoc comparisons confirmed that adjacent energy levels are frequently indistinguishable. Diagnostic assessability did not differ significantly across 60 to 90 keV in calcified plaque or across 40 to 80 keV in non-calcified components, and artifact ratings in non-calcified components did not differ significantly between any two energy levels. No energy level, however, achieved higher ratings than 70 to 80 keV for calcified plaque, or than 40 to 50 keV for non-calcified components, on all three scales simultaneously.

**Figure 3:**
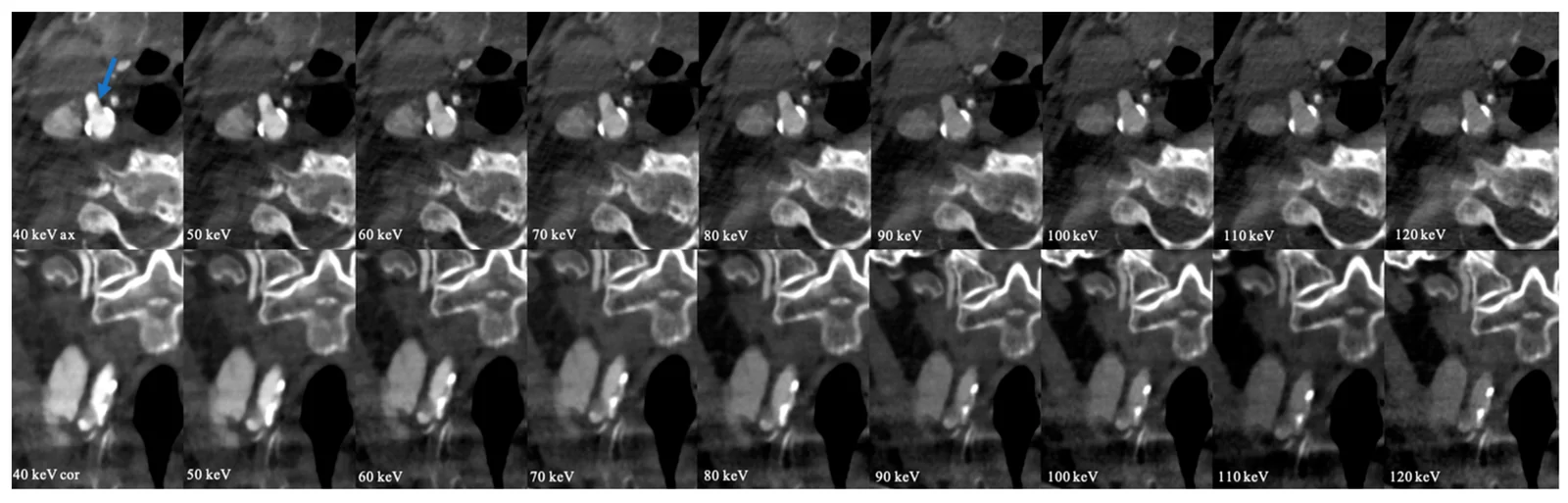
Photon-counting CT (PCCT) virtual monoenergetic images (VMI) of a patient with right-sided carotid atherosclerotic disease. Axial (**top row**) and coronal (**bottom row**) reconstructions from 40 to 120 keV. At low keV (40–50 keV), the soft plaque at the carotid bifurcation (blue arrow) is more conspicuous due to increased vascular contrast, but blooming and beam-hardening artifacts from the adjacent calcified plaque are more pronounced, limiting stenosis assessment. At higher keV (70–80 keV), these artifacts are reduced, improving delineation of calcified plaque borders and the vascular lumen.

A summary of qualitative scores for image quality, artifact burden, and diagnostic assessability across energy levels is presented in Table 4. Representative images illustrating the effect of keV variation on plaque depiction are shown in Figure 3.

## 4. Discussion

This study systematically evaluated the potential of PCCT-derived VMI for the depiction of carotid atherosclerosis, focusing on image quality and diagnostic assessability, and in particular on adapting the energy level to plaque composition. Quantitative analyses showed that calcification-related artifacts declined progressively with increasing keV, while iodine contrast decreased in parallel. The reader assessment identified 70–80 keV as the best compromise for calcified plaque and 40–50 keV for non-calcified components.

Quantitatively, attenuation and CNR were highest at low keV levels (40–50 keV), maximizing vascular contrast. Corrected image noise and AIX were also greatest in this range, so the gain in contrast came at the cost of the highest noise and artifact burden. With increasing keV, both declined progressively, and by 70–80 keV, AIX had fallen by roughly half relative to 40 keV while attenuation remained well above soft-tissue values. SNR did not follow this pattern and reached its highest values only at 110–120 keV. This trade-off highlights the limitations of applying a universal low-keV reconstruction (40–55 keV), as suggested by the literature, to heavily calcified arteries and supports a more differentiated, composition-specific approach [20,22,28].

The divergent behavior of SNR and CNR reflects the manner in which noise is propagated in virtual monoenergetic reconstruction. VMI datasets are synthesized from material-basis images, and at low keV, the synthesis is weighted towards the photoelectric basis component, so that both the iodine signal and the variance of the basis images are amplified together. Above approximately 70 to 80 keV, the reconstruction converges towards the Compton-dominated basis image, in which attenuation is only weakly energy-dependent, so noise falls onto a low plateau while the residual signal declines more gradually. Because SNR is referenced to the standard deviation of subcutaneous fat, it is governed almost entirely by this noise behavior, whereas the numerator of CNR is the artery-to-muscle difference, which is driven by iodine. Although SNR was highest at 110 to 120 keV, this reflects the collapse of image noise rather than a diagnostic advantage, since intraluminal attenuation at 120 keV (172.30 ± 9.40 HU) approaches that of the surrounding soft tissue and the lumen can no longer be reliably delineated. SNR alone is therefore not a suitable criterion for energy-level selection in CT angiography. 

The monotonic decline of AIX alongside a subjective artifact optimum at 80 keV merits comment. AIX is derived from the difference in the standard deviations of two regions of interest and therefore expresses the dispersion of streak and blooming artifacts in absolute Hounsfield units. Because overall image contrast compresses as keV rises, this absolute dispersion necessarily decreases even when the artifact is unchanged relative to its surroundings. Above 80 keV, the additional reduction in AIX was small (48.53 ± 39.39 at 80 keV versus 38.96 ± 34.29 at 120 keV), whereas intraluminal attenuation fell by more than half over the same range. Since readers judge artifact burden in relation to the lumen they are delineating, a marginal absolute gain in artifact suppression is not perceived as beneficial when accompanied by a substantial loss of vascular contrast. Blooming is moreover largely an edge phenomenon determined by the system point-spread function and partial-volume averaging, which a variance-based index reflects only in part.

The qualitative findings underscore the importance of tailoring energy-level selection to plaque composition. Radiologists rated non-calcified plaques most favorably at 40 and 50 keV, near the K-edge enhancement of iodine. Calcified plaques were best evaluated at 70 and 80 keV, where diagnostic assessability was highest owing to improved lumen visualization and reduced artifact burden. The present findings indicate that lower-keV reconstructions improve visualization of non-calcified plaque but are less suitable for the assessment of heavily calcified lesions because of increased blooming artifacts. Consequently, higher-keV reconstructions provide a more favorable depiction of calcified carotid plaque.

Iodine demonstrates a K-edge at 33.2 keV, producing a pronounced increase in attenuation near this energy level and thereby enhancing contrast in low-keV reconstructions. Calcium lacks a K-edge within the diagnostic energy range yet maintains a relatively high attenuation coefficient at low keV values owing to its higher atomic number and density. Consequently, although both materials demonstrate increased attenuation at lower keV, calcium tends to dominate image brightness at these levels, contributing to blooming artifacts and potentially obscuring vessel lumens in heavily calcified segments [29,30]. 

In comparison with previous studies, our findings both align with and extend the current evidence on VMI at varying keV levels. The individual elements of this approach are not new. Higher-energy reconstruction has been shown to improve the depiction of calcified carotid plaque in dual-energy CT, with 80–100 keV corresponding most closely to histology [31], and the full virtual monoenergetic range has been characterized in the carotid arteries using dual-layer systems, both for vessel contrast and plaque depiction [32] and for plaque burden [33]. In PCCT, the dependence of plaque component volumes on energy level has been demonstrated in the coronary arteries [34], composition-specific energy selection has been explicitly proposed as a strategy in spectral cardiac CT [35], and carotid plaque and stenosis have recently been evaluated across monoenergetic and calcium-removal reconstructions [36]. Our own earlier work characterized low-energy reconstruction in the carotid arteries [22]. What the present study adds is the quantification of calcification-related artifacts (corrected artifact burden and artifact index) as a function of energy level in the carotid arteries on a dual-source photon-counting system and a reader comparison of the calcified and non-calcified components at each energy level [20,22,25]. 

Virtual calcium removal represents an alternative approach to the same problem. Bierens et al. found stenosis measurements to be stable across energy levels, with potential advantages of the calcium-removal technique [36]. Such algorithms may in future prove superior to energy-level selection for heavily calcified lesions. Virtual monoenergetic reconstructions are nevertheless available on every spectral platform and require no additional postprocessing, so composition-adapted energy selection remains of practical relevance where dedicated calcium-removal reconstructions are not implemented.

Beyond image quality and diagnostic assessability, these findings have important practical implications for clinical routine. Firstly, plaque-specific keV optimization may save time and data storage. Rather than reconstructing the entire VMI spectrum, selecting only the most favorable energy levels (40–50 keV for non-calcified plaques and 70–80 keV for calcified plaques) can reduce computational load and potentially shorten reconstruction time, facilitating leaner imaging protocols without compromising clinical information. Second, the proposed approach is readily implementable in clinical workflows. Once plaque composition has been identified, either visually or through automated plaque classification algorithms in the future, radiologists can directly access the corresponding keV reconstruction with the highest expected diagnostic yield, which may streamline interpretation. With future integration of artificial-intelligence tools, automated plaque detection and keV suggestion could further increase efficiency and standardization across institutions. This strategy aligns with recommendations from the European Society of Radiology and the European Society for Vascular Surgery, which advocate for individualized imaging protocols adapted to patient and lesion characteristics [37]. Finally, standardizing plaque-specific keV protocols could improve longitudinal follow-up by enabling more reliable detection of subtle changes in plaque morphology, thereby supporting personalized treatment planning and monitoring.

Several limitations should be acknowledged. First, the design was retrospective and single-center, and the cohort was derived from patients undergoing pre-TAVI CTA, which may limit generalizability to the broader population with carotid disease. Second, no subgroup analyses by cardiovascular risk profile were performed, as this was not among the predefined aims of the study. Chronic kidney disease promotes medial arterial calcification, and the interaction between renal function, calcification burden, and the optimal energy level warrants dedicated prospective investigation. Third, the classification used here is not a measurement of plaque composition. Histopathology remains the reference standard, and multicontrast vessel-wall MRI is the preferred non-invasive modality for the lipid-rich necrotic core, intraplaque hemorrhage, and fibrous-cap status. CT reliably identifies calcification, but the attenuation ranges of fibrous tissue, lipid-rich core, and hemorrhage overlap substantially, and iodine within the lumen compounds this overlap in contrast-enhanced acquisitions. Our binary categorization therefore separates lesions that produce calcium-related blooming from those that do not, which is sufficient for energy-level selection. Fourth, this study assessed image quality and diagnostic assessability rather than stenosis grading. Because no standardized stenosis measurements or independent reference standard were applied, the findings should not be interpreted as demonstrating improved diagnostic accuracy for stenosis severity. Fifth, longitudinal clinical outcomes were not assessed. Future prospective multicenter studies should investigate how keV selection affects clinical decision-making and patient outcomes such as stroke prevention, ideally incorporating a stenosis reference standard, formal reader-agreement analysis, and integration with radiomics or machine-learning models. Sixth, no washout interval was interposed between energy levels in the qualitative assessment, as this would have required nine separate reading sessions for every patient. Recall effects can therefore not be entirely excluded. Finally, a conventional polyenergetic reconstruction was not analyzed as a separate comparator, with the 120 keV virtual monoenergetic series serving as the high-energy reference.

## 5. Conclusions

In summary, the reader-rated image quality, artifact burden, and diagnostic assessability of carotid plaque on PCCT depend on plaque composition, with 40 to 50 keV most favorable for non-calcified and 70 to 80 keV for calcified components, and the quantitative behavior of vascular contrast and of calcium-related artifacts across the energy range is consistent with this. These findings support dual-range reconstruction at 40 to 50 and 70 to 80 keV as a protocol default. They concern image quality and plaque depiction only; whether composition-adapted energy selection improves the accuracy of stenosis grading remains to be established in studies using standardized stenosis measurements against an independent reference standard.

## Figures and Tables

**Table 1:** Baseline characteristics of the 111 included patients. SD, standard deviation.

Characteristic	Value
Age (years), mean ± SD (min; max)	80 ± 7.5 (56; 93)
Male sex, *n* (%)	64 (57.7)
Female sex, *n* (%)	47 (42.3)
Body mass index (kg/m^2^), mean ± SD (min; max)	28 ± 5.0 (18; 44)
Peripheral arterial disease, *n* (%)	26 (23.4)
Arterial hypertension, *n* (%)	105 (94.6)
Dyslipidemia, *n* (%)	87 (78.4)
Chronic kidney disease, *n* (%)	61 (55)
Diabetes mellitus, *n* (%)	25 (22.5)
Current smokers, *n* (%)	6 (5.4)
Former smokers, *n* (%)	24 (21.6)
Non-smokers, *n* (%)	48 (43.3)
Smoking status unknown, *n* (%)	33 (29.7)

**Table 2:** Acquisition and reconstruction parameters.

Parameter	Value
Scanner	NAEOTOM Alpha, dual-source photon-counting detector (Siemens Healthineers, Forchheim, Germany)
Scan direction	Craniocaudal
Tube voltage	120 kV
CARE keV image quality level	85
Detector collimation	2 × 144 × 0.4 mm
Rotation time	250 ms
Pitch	3.2
Field of view	350 mm
Matrix	512 × 512
Slice thickness/increment	1.0 mm/1.0 mm
Convolution kernel	Qr40
Quantum iterative reconstruction	QIR strength 3
VMI reconstruction	40–120 keV in 10 keV increments (syngo.via VB60A)
Contrast medium	80 mL Xenetix 350 (iobitridol 76.78 g/100 mL; Guerbet, Villepinte, France)
Injection rate	5 mL/s, followed by 50 mL saline at the same rate
Venous access	20-gauge cannula, antecubital fossa
Bolus tracking	Region of interest in the descending aorta, threshold 100 HU, 5 s delay
ECG synchronization	40% of the RR interval

CARE keV, automated tube voltage and quality reference selection; VMI, virtual monoenergetic imaging; QIR, quantum iterative reconstruction; HU, Hounsfield units.

**Table 3:** Results of the quantitative measurements (mean ± SD). The *p*-value refers to the comparison of each parameter across all energy levels (Friedman test). keV, kiloelectronvolt; HU, Hounsfield units; AIX, artifact index; CIN, corrected image noise; CNR, contrast-to-noise ratio; SNR, signal-to-noise ratio; SD, standard deviation.

keV	HU	AIX	CIN	CNR	SNR	*p*-Value
40	1387.10 ± 70.55	112.46 ± 99.07	76.14 ± 91.98	42.00 ± 21.91	42.58 ± 21.55	<0.0001
50	924.86 ± 46.48	82.74 ± 70.44	58.88 ± 65.90	39.87 ± 20.94	41.04 ± 20.83	<0.0001
60	642.99 ± 32.25	65.66 ± 53.29	48.19 ± 50.47	37.16 ± 19.13	39.19 ± 19.39	<0.0001
70	468.94 ± 23.46	55.29 ± 44.17	41.25 ± 41.69	35.98 ± 19.44	38.86 ± 17.78	<0.0001
80	358.69 ± 17.21	48.53 ± 39.39	36.69 ± 37.65	37.60 ± 29.66	41.16 ± 18.91	<0.0001
90	285.31 ± 24.46	44.99 ± 36.99	34.43 ± 35.61	38.98 ± 41.47	37.84 ± 21.47	<0.0001
100	234.30 ± 11.54	43.43 ± 36.69	33.78 ± 35.27	38.84 ± 47.80	44.67 ± 22.07	<0.0001
110	198.34 ± 10.21	40.44 ± 34.93	32.32 ± 33.71	38.12 ± 51.02	45.59 ± 23.47	<0.0001
120	172.30 ± 9.40	38.96 ± 34.29	31.81 ± 33.16	36.80 ± 52.41	46.05 ± 24.83	<0.0001

**Table 4:** Results of the qualitative assessment, reported as the mean of the two readers’ five-point Likert ratings, separately for calcified and non-calcified plaques across energy levels.

keV	Overall Image Quality	Artifacts	Diagnostic Assessability	Kappa Value
40 keV	3.8 ± 0.7	3.4 ± 0.9	3.5 ± 0.9	0.82 (95% CI 0.74–0.88)
50 keV	3.8 ± 0.7	3.6 ± 0.8	3.7 ± 0.8	0.84 (95% CI 0.77–0.89)
60 keV	4.1 ± 0.6	4.1 ± 0.7	4.0 ± 0.8	0.88 (95% CI 0.83–0.92)
70 keV	4.4 ± 0.5	4.3 ± 0.6	4.2 ± 0.7	0.91 (95% CI 0.87–0.95)
80 keV	4.4 ± 0.5	4.5 ± 0.5	4.2 ± 0.7	0.92 (95% CI 0.88–0.96)
90 keV	4.2 ± 0.6	4.3 ± 0.6	3.9 ± 0.8	0.90 (95% CI 0.85–0.94)
100 keV	4.2 ± 0.6	4.3 ± 0.6	3.5 ± 0.9	0.88 (95% CI 0.82–0.92)
110 keV	4.0 ± 0.7	4.2 ± 0.7	3.2 ± 1.0	0.84 (95% CI 0.77–0.89)
120 keV	3.7 ± 0.8	4.2 ± 0.7	3.0 ± 1.0	0.80 (95% CI 0.71–0.87)
40 keV	4.4 ± 0.5	4.2 ± 0.7	4.4 ± 0.5	0.88 (95% CI 0.82–0.93)
50 keV	4.4 ± 0.5	4.2 ± 0.7	4.4 ± 0.5	0.89 (95% CI 0.84–0.93)
60 keV	4.2 ± 0.6	4.2 ± 0.7	4.2 ± 0.6	0.87 (95% CI 0.81–0.92)
70 keV	4.1 ± 0.6	4.1 ± 0.7	4.2 ± 0.6	0.85 (95% CI 0.78–0.90)
80 keV	4.1 ± 0.7	4.1 ± 0.7	4.2 ± 0.6	0.84 (95% CI 0.77–0.90)
90 keV	4.0 ± 0.7	4.0 ± 0.8	3.8 ± 0.8	0.82 (95% CI 0.74–0.89)
100 keV	3.8 ± 0.8	4.0 ± 0.8	3.8 ± 0.8	0.80 (95% CI 0.71–0.87)
110 keV	3.7 ± 0.8	4.0 ± 0.8	3.3 ± 0.9	0.77 (95% CI 0.67–0.85)
120 keV	3.5 ± 0.9	4.0 ± 0.8	3.0 ± 1.0	0.74 (95% CI 0.63–0.83)

Values are the mean ± standard deviation of the two readers’ five-point Likert ratings (*n* = 111 lesions). The kappa value is the quadratically weighted Cohen’s kappa for inter-reader agreement at that energy level, with its 95% confidence interval.

## Data Availability

The data presented in this study are available on request from the corresponding author. The data are not publicly available due to data protection.

## Abbreviations

AIX	Artifact index
CIN	Corrected image noise
CNR	Contrast-to-noise ratio
CTA	CT angiography
HU	Hounsfield units
PCCT	Photon-counting CT
ROI	Region of interest
SD	Standard deviation
SNR	Signal-to-noise ratio
VMI	Virtual monoenergetic images
